# Workplace harassment and violence in the public sector: an integrative literature review

**DOI:** 10.47626/1679-4435-2026-1537

**Published:** 2026-02-27

**Authors:** Luana Gonçalves De Vito, Marli Aparecida Reis Coimbra, Rafaela Cristina Sanfelice

**Affiliations:** 1 Universidade Federal do Triângulo Mineiro, Departamento de Atenção à Saúde do Servidor, Uberaba, MG, Brazil; 2 Universidade Federal de Alfenas, Instituto de Ciência e Tecnologia, Alfenas, MG, Brazil

**Keywords:** public sector, non-sexual harassment, workplace violence, psychosocial impact, aggression., setor público, assédio não sexual, violência no trabalho, impacto psicossocial, agressão.

## Abstract

Workplace violence is associated with workers’ mental health impairment. This study aimed to characterize the scientific production on workplace harassment and/or violence in the public sector and to synthesize the main available evidence. An integrative literature review was conducted, with a protocol registered on the Open Science Framework platform (https://osf.io/phq4z/). Searches were performed in the VHL, PubMed, and APA PsycInfo^®^. The search strategy included the descriptors “public sector,” “harassment, non-sexual,” and “workplace violence.” Inclusion criteria were original articles published within the last 10 years, available in full text, written in Portuguese, English, or Spanish, whose study population included public sector workers and addressed workplace harassment and/or violence. After the selection process, 10 articles were included. Verbal violence showed a prevalence ranging from 19.4% to 63.5%, followed by physical violence, with rates between 9.8% and 16%, and sexual harassment, ranging from 9.1% to 12%. The public sector was approximately three times more affected compared with the private sector. Factors associated with increased susceptibility to workplace harassment and violence included being married, female sex, working as a physician or member of the nursing staff, night shift work, and professional practice in hospital emergency departments. Managerial support was identified as a protective factor. Repeated exposure to violent episodes was associated with an increased risk of suicide attempts and death by suicide. Public sector workers are significantly exposed to workplace harassment and violence. Greater attention from managers is recommended, along with strengthening public policies and educational programs aimed at implementing preventive measures and promoting healthier work environments.

## Abstract (pt)

A violência no ambiente de trabalho está associada ao adoecimento mental dos trabalhadores. O objetivo deste estudo foi caracterizar a produção científica sobre assédio moral e/ou violência laboral no setor público e sintetizar as principais evidências disponíveis. Trata-se de uma revisão integrativa da literatura, com protocolo registrado na plataforma Open Science Framework (https://osf.io/phq4z/). A busca foi realizada nas bases de dados BVS, PubMed e APA PsycInfo^®^. Para a estratégia de busca, foram utilizados os descritores “public sector”, “harassment, non-sexual”, “workplace violence”. Os critérios de inclusão foram: artigos originais publicados nos últimos 10 anos, disponíveis na íntegra, nos idiomas português, inglês ou espanhol, cuja população de estudo incluísse trabalhadores do setor público e abordasse a temática do assédio moral e/ou da violência no trabalho. Ao final do processo de seleção, 10 artigos foram incluídos. A violência verbal apresentou prevalência entre 19,4% e 63,5%, seguida da violência física, com taxas entre 9,8% e 16%, e do assédio sexual, entre 9,1% e 12%. Observou-se que o setor público foi aproximadamente três vezes mais acometido em comparação ao setor privado. Entre os fatores associados à maior predisposição ao assédio moral e à violência no trabalho destacaram-se: estado civil casado, sexo feminino, atuação como médico ou enfermeiro, trabalho em turno noturno e exercício profissional em setores de emergência hospitalar. O apoio do gestor foi identificado como fator protetor. A ocorrência repetida de episódios de violência esteve associada ao aumento do risco de tentativa e morte por suicídio. Conclui-se que trabalhadores do setor público estão expostos de forma significativa ao assédio moral e à violência no ambiente laboral. Recomenda-se maior atenção por parte de gestores, bem como o fortalecimento de políticas públicas e programas educativos voltados à implementação de medidas preventivas e à promoção de ambientes de trabalho mais saudáveis.

## INTRODUCTION

The World Health Organization (WHO) has reported that approximately 1 billion people have experienced mental disorders, and about 15% of working-age adults have also dealt with these conditions in recent years. Multiple factors contribute to impaired mental health, among which the work environment stands out as a key determinant, as it may intensify social problems, particularly bullying and psychological violence, which are often associated with workplace harassment and have significant effects on psychological well-being ^[1]^.

According to a global survey conducted by the International Labour Organization (ILO), nearly 23% of workers, approximately one in five employees, reported having experienced violence and harassment in the workplace ^[2]^.

ILO Convention No. 190 ^[2]^, together with Recommendation No. 206, represents the first set of international standards specifically aimed at preventing, addressing, and reducing violence and harassment at work. These instruments provide a normative framework for addressing the issue and, for the first time in international law, recognize the right of all individuals to a respectful work environment. They also establish the responsibility of States to ensure the implementation of measures to prevent such practices ^[3]^.

The defining characteristic of workplace harassment is the prolonged and frequent exposure of one or more individuals to humiliating or constraining situations, either directly or indirectly, resulting in significant harm. Indirect forms include persecution, work overload, dissemination of rumors, and social isolation. Direct forms include accusations, public humiliation, and verbal aggression perpetrated by the harasser ^[4]^.

Public administration has structural features that make it particularly susceptible to workplace harassment. Job stability, difficulty in dismissing employees, and formal evaluation and control processes may contribute to the development of hostile environments. Consequently, workplace harassment and violence may emerge as indirect forms of punishment or retaliation, in which perpetrators use institutional mechanisms to promote isolation and pressure workers, including excessive demands related to deadlines and targets, often without adequate mental health support ^[5]^.

The absence of clear prevention and response policies, combined with internal power disputes, increases employee vulnerability, while impunity for perpetrators contributes to the persistence of these practices. In addition, patronage relationships and favoritism, present in some sectors of public administration, may reinforce a context in which workplace harassment and violence are used as mechanisms of exclusion or marginalization ^[5]^.

This study addresses workplace harassment among public sector workers, considering its intersection with work, health, and well-being as individual rights and guarantees. Workplace harassment and violence compromise quality of life, contribute to the development of mental disorders, and reduce work productivity ^[6]^.

Furthermore, as established by Regulatory Standard No. 1 (NR-1), issued by the Ministry of Labour and Employment, organizations must assess work-related psychosocial risks. These risks, linked to work organization and interpersonal relationships, directly affect workers’ well-being, increase stress levels, and contribute to the onset of mental disorders. Factors such as lack of institutional support, workplace harassment, and interpersonal conflicts are frequently associated with anxiety and depression ^[6]^.

Psychosocial risks directly affect occupational health and safety, and NR-1 establishes the need for their recognition and control ^[6]^. Therefore, the objective of this study was to characterize the scientific production on workplace harassment and/or violence in the public sector and to synthesize the main available evidence.

## METHODS

### STUDY DESIGN AND PROTOCOL

This study is an integrative literature review ^[7]^, with the review protocol registered on the Open Science Framework platform (https://osf.io/phq4z/) under DOI 10.17605/OSF.IO/PHQ4Z.

### STAGES OF THE INTEGRATIVE REVIEW

The review was conducted in six stages, as described in literature ^[7]^: i) identification of the topic and formulation of the review question; ii) development of the search strategy, definition of inclusion and exclusion criteria, and database search; iii) data extraction from selected studies; iv) critical appraisal of eligible studies; v) analysis and interpretation of findings; and vi) presentation of the review.

### REVIEW QUESTION

The review question was: “What are the characteristics of the scientific production on workplace harassment and/or workplace violence in the public sector?” This question was structured using the PICo strategy, in which P (population) refers to public sector workers; I (phenomenon of interest) refers to the scientific production on workplace harassment and/or workplace violence in this context; and Co (context) refers to exposure to workplace harassment and violence in the work environment.

### ELIGIBILITY CRITERIA

Original articles published between 2014 and 2024, available in full text in electronic format, written in Portuguese, English, or Spanish, and investigating workplace harassment and/or workplace violence in public sector settings were included. Duplicate articles, editorials, opinion and review articles, letters, comments, notes, theses, dissertations, and manuals were excluded ^[7,8]^.

### INFORMATION SOURCES AND SEARCH STRATEGY

Data collection was conducted between November and December 2024 using the following databases: VHL, PubMed, and APA PsycInfo^®^.

The search strategy was developed following consultation with the Health Sciences Descriptors, selecting the terms “public sector,” “harassment, non-sexual,” and “workplace violence.” Corresponding descriptors were also identified in Medical Subject Headings and the PsycINFO Thesaurus.

Search strategies were structured by combining descriptors and their synonyms using the Boolean operators OR and AND, considering the specific features of each database. The final search strategy is presented in Table 1.

**Table 1:** Search strategy across databases

Database	Search string
VHL	*Public health service OR public sector* ANDHarassment, non-sexual OR behavior OR aggression OR crime OR violence OR bullying ANDWorkplace violence
PubMed	*United States public health service OR public sector* AND *Harassment, non-sexual OR behavior OR aggression OR crime OR violence* AND *Workplace violence*
APA PsycInfo^®^	*Government employees OR public sector* AND *Harassment, non-sexual OR behavior OR aggression OR crime OR violence OR bullying* AND *Workplace violence*

Source: Prepared by the authors (2025).

### STUDY SELECTION

After the searches were completed, the identified articles were imported into Zotero^®^ for reference storage and management, and duplicates were removed. Subsequently, two review authors independently screened titles and abstracts, followed by full-text review, according to the previously established eligibility criteria ^[7]^.

### DATA EXTRACTION AND ANALYSIS

Data extracted for critical analysis were organized using an instrument widely applied in integrative reviews, including the following items: title, authors, year and country of publication, study objective, and main findings (results and recommendations) ^[8,9]^. The studies were also assessed for methodological rigor using the same instrument ^[9]^.

## RESULTS

A total of 109 articles were identified across the selected databases. Of these, two were excluded due to duplication, retaining only one version of each study. After title and abstract screening, 95 articles did not meet the inclusion criteria. Consequently, 12 studies were selected for full-text review. Following this stage, two studies did not meet the eligibility criteria, resulting in a final sample of 10 articles included in this integrative review.

**Figure 1:**
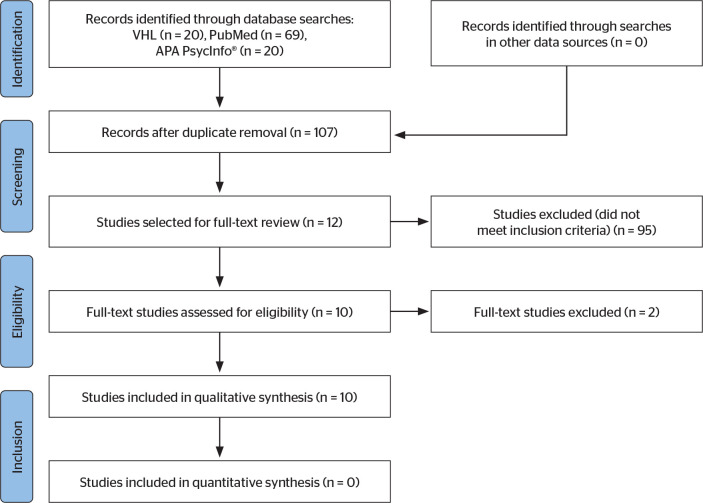
Preferred Reporting Items for Systematic Reviews and Meta-Analyses flowchart illustrating the study search and selection process conducted by the reviewers.

Regarding database distribution, six articles were retrieved from the VHL, three from APA PsycInfo^®^, and one from PubMed. Figure 1 presents the flowchart of the search and study selection process according to the Preferred Reporting Items for Systematic Reviews and Meta-Analyses guidelines ^[10]^.

The studies included in this review were conducted across different continents. Countries in Asia (Taiwan, Bangladesh, and Jordan) and Europe (Finland) accounted for 33.3% of the total, followed by countries in Africa (Nigeria and Ghana) and South America (Brazil and Chile), each representing 22.2%. A quantitative cross-sectional design predominated, present in 80% of the studies.

Verbal violence was the most frequent form of aggression (19.4%-63.5%), followed by physical violence (9.8%-16%) and sexual harassment (9.1%-12%).

Women represented most participants (53.8%), and the predominant age range was between 20 and 44 years (59.8%). Approximately 48.9% of participants reported being married. Most workers had between 6-10 years of experience in emergency services (55.2%).

The public sector settings examined in the included studies were predominantly public hospitals. Accordingly, the study populations consisted of physicians, paramedics, nursing staff, service assistants, and administrators, with medical and nursing teams representing the largest groups.

Table 2 presents the characterization of the scientific production, including title, authors, year and country of publication, objectives, and main results and recommendations.

**Table 2:** Characterization of the scientific production

Title, authors, year, and country	Objective	Results/recommendations
Acoso laboral y factores asociados en trabajadores de servicios de emergencias Paravic-Klijn et al. ^[11]^, 2018, Chile	To determine workplace harassment and associated factors among workers in emergency services of public and private health facilities.	Workplace harassment prevalence was three times higher in the public sector than in the private sector, particularly among women and single workers. Perpetrators were most often coworkers, followed by supervisors. The study highlights the need for measures to address workplace harassment.
Determinants of workplace violence against clinical physicians in hospitals Wu et al. ^[12]^, 2015, Taiwan	To examine organizational determinants of workplace violence against clinical physicians.	Overall, 41.5% reported at least one threat of physical or verbal violence, and 9.8% reported sexual harassment in the previous 3 months. Higher rates were associated with greater work demands and perceived insecurity.
Prevalence of Psychological Workplace Violence among Employees of a Public Tertiary Health Facility in Enugu, Southeast Nigeria Chinawa et al. ^[13]^, 2020, Nigeria	To determine the prevalence of psychological workplace violence among employees in a tertiary health institution in Nigeria.	The study showed a high prevalence of psychological violence (49.7%). Verbal abuse (40.8%), bullying (7.0%), and sexual harassment (1.9%) were the most reported forms. Most perpetrators were patients’ relatives, while 23.4% were staff members. In 28.6% of cases, no action was taken, and only 1% were formally reported. Bullying was markedly higher among health professionals.
Sources, incidence and effects of non-physical workplace violence against nurses in Ghana Boafo et al. ^[14]^, 2016, Ghana	To document the incidence, sources, and effects of verbal abuse and sexual harassment against Ghanaian nurses.	Results showed that 52.2% experienced verbal abuse, mostly perpetrated by patients’ relatives (45.5%), and 12% experienced at least one episode of sexual harassment, most frequently perpetrated by physicians (50%). Chi-square testing showed significant associations between gender, workplace violence, and intention to leave nursing. Reported consequences ranged from distressing memories to constant vigilance. The study recommended educational programs and clear policies to increase awareness of the problem.
Violência no trabalho: um estudo com servidores públicos da saúde Silva et al. ^[15]^, 2014, Brazil	To investigate workplace violence as a factor influencing the health of public health workers.	The study found that 25.9% experienced at least 1 form of workplace violence, with verbal aggression being the most common (19.4%). Workplace harassment prevalence was 10.5%. Findings highlight the importance of increasing visibility of violence in the health sector and supporting policies focused on worker health.
Workplace violence among nursing professionals Bernardes et al. ^[16]^, 2021, Brazil	To identify types of occupational violence experienced by nursing staff.	Approximately 88.9% reported workplace violence, with workplace harassment among the most cited occurrences (25.4%), followed by physical violence (11%) and sexual harassment (9.1%). Most participants (90%) believed incidents could have been prevented. Violence was attributed to patients, relatives, coworkers, and supervisors, reinforcing the need for preventive measures to promote workplace safety.
Effectiveness of a workshop-based intervention to reduce bullying and violence at work: A 2-year quasi-experimental intervention study Seppälä et al. ^[17]^, 2023, Finland	To develop and examine the effectiveness of an intervention aimed at preventing bullying and workplace violence through psychosocial work environment modifications.	No direct or indirect effects on bullying or workplace violence were identified. However, intervention group B showed improvements in perceived support and organizational justice compared with the control group. These findings were not replicated in intervention group A. The study suggested refining the intervention and prioritizing interpersonal relationships and team psychosocial resources.
Violence against physicians in Jordan: An analytical cross-sectional study Alhamad et al. ^[18]^, 2021, Jordan	To explore workplace violence within the medical community, analyze the characteristics of reported abuse cases in terms of perpetrators, timing, type of abuse, and consequences, and identify an effective role-based approach to address the growing problem.	Results showed that 63.1% of physicians experienced workplace violence in the previous year. Violence was more frequent among physicians in the governmental sector. Among public sector physicians (n = 253), 75.3% reported violence, including verbal aggression (63.5%) and physical aggression (10.4%). Only 13.3% felt legally protected. Anti-violence actions were recommended.
Workplace Violence Among Health Care Professionals in Public and Private Health Facilities in Bangladesh Shahjalal et al. ^[19]^, 2021, Bangladesh	To examine the prevalence of workplace violence and its associated factors and to explore the experiences of health professionals.	Results showed that among professionals exposed to workplace violence (43%, n = 468), 84% reported non-physical violence and 16% reported physical violence. In addition, 65% stated that no investigative measures were taken, and 44% reported no consequences for perpetrators. Approximately 51.4% of physicians and 35.4% of nursing staff were exposed to some form of violence. Workplace violence was significantly associated with four factors: being married, working in the public sector, working in emergency settings, and shift work. The study concluded that there is an urgent need to establish formal guidelines for reporting and managing workplace violence.
Association of workplace violence and bullying with later suicide risk: a multicohort study and meta-analysis of published data Hanson et al. ^[20]^, 2023, Finland	To evaluate the association between workplace violence and bullying and the risk of suicide attempts and death in multicohort studies.	A total of 1,103 suicide attempts or deaths were recorded among 205,048 participants with workplace violence data. The results indicated that Finnish public sector employees (28%) were exposed to workplace violence at higher rates compared with other study populations. Workplace violence was associated with an increased risk of suicide, particularly among individuals exposed more frequently. The study highlights the importance of preventing violent behavior in the workplace.

Source: Prepared by the authors (2025).

## DISCUSSION

This review found that workplace harassment in the public sector represents a relevant and concerning issue. This sector requires particular attention in the assessment of psychosocial work risks, as workplace harassment ^[11]^ is associated with the development of mental disorders throughout professional trajectories ^[6]^. The public sector showed an approximately threefold higher prevalence of this risk compared with the private sector as well as greater susceptibility among women and single individuals ^[11]^. Women were particularly vulnerable to workplace and/or sexual harassment, a condition associated with social and cultural factors that contribute to mental health impairment ^[21]^.

The studies included in this review demonstrated that workplace harassment and violence are frequent situations that require greater attention ^[11,12]^. In one study, 41.5% of participants reported receiving at least one threat of physical or verbal violence ^[12]^. In another, psychological violence showed a prevalence of 49.7%, followed by verbal abuse (40.8%), bullying (7.0%), and sexual harassment (1.9%) ^[13]^. These events were mainly associated with work demands and a perceived climate of insecurity in the workplace ^[12]^. The high frequency of these occurrences reinforces the need for reflection and implementation of preventive measures and appropriate institutional responses.

The main perpetrators identified were coworkers, managers ^[11]^, and relatives of patients treated by health professionals ^[13,14]^. A substantial proportion of professionals exposed to workplace violence did not take any action after these episodes (28.6%) ^[13]^, contributing to worsening psychological impacts, including persistent fear, intense psychological distress, burnout syndrome ^[22]^, and an increased risk of suicide attempts and death over the course of a career ^[23]^. These findings highlight the need for more effective institutional actions.

Among the studies analyzed, research conducted with nurses in Ghana reported a high prevalence of verbal harassment (52.2%), mostly perpetrated by patients’ relatives, whereas sexual harassment was most frequently perpetrated by physicians (50%) ^[14]^. Reported consequences included recurrent traumatic memories, constant vigilance, and intention to leave the profession ^[14]^, demonstrating significant psychological impacts on professional trajectories.

The WHO and the ILO emphasize, in a joint report, the need to investigate the causes of work-related illness ^[24]^. The studies included in this review identify workplace harassment and violence as features present across different professional contexts and reinforce the need to implement practices focused on awareness and response to the problem ^[11,14]^.

Health professionals working in public services experience workplace harassment and/or violence in daily practice, requiring specific institutional attention and measures ^[15]^. In emergency settings, 61.7% of reported violence was attributed to perpetrators in these environments ^[25]^. This high prevalence may be related to direct exposure to situations of suffering and tension. Female sex and night shift work were also identified as vulnerability factors ^[25]^.

Another study included in this review reported that 88.9% of nursing professionals in a public hospital emergency unit experienced workplace harassment. In addition to verbal, physical, and sexual violence, episodes of racial discrimination were also recorded ^[16]^. Verbal violence emerged as the most frequent form of aggression ^[13,14,16]^, highlighting the need for improved institutional preparedness in conflict management and promotion of nonviolent communication. High levels of underreporting and stigma were also observed, associated with the low formal reporting of occupational violence ^[16]^.

Among physicians, a higher prevalence of workplace harassment was observed among men compared with women (67.2% vs 55.3%) ^[18]^. In this group, men reported higher frequencies of verbal and physical aggression and weapon-related attacks, whereas sexual harassment was more frequently reported by women ^[18]^, reflecting sociocultural aspects related to gender dynamics.

Workplace violence among physicians and nursing staff was associated with factors such as marital status (married), employment in the public sector, work in emergency services, and shift work ^[19]^. Additionally, leaving the profession due to workplace violence was identified in some studies included in this review ^[14,19]^.

Occupational violence and bullying, when repeated and persistent, were associated with increased risk of suicide attempts and death among workers in public hospitals ^[20]^. These findings reinforce the importance of expanding research on the topic and implementing institutional policies aimed at awareness, protection, and mental health promotion. The need for psychological support, strengthening of formal guidelines, and improvement of interpersonal relationships in the workplace was also highlighted ^[11,18-20]^.

Workplace harassment and violence can be understood as important psychosocial risk factors, as they represent concrete situations that contribute to negative consequences for workers’ mental health and well-being. Recurrent humiliation, excessive demands, exclusion, and disqualification weaken individuals and increase the likelihood of stress, anxiety, depression, and interpersonal conflict. The absence of protective factors, such as organizational support and role clarity, intensifies these effects and contributes to the persistence of work environments more vulnerable to violence ^[26]^.

Mental disorders have a significant impact on work absenteeism, and when recurrent, recovery tends to be more complex and prone to relapse. In this context, the work environment plays a decisive role: settings marked by harassment, bullying, or excessive pressure contribute to clinical deterioration, whereas supportive attitudes from supervisors help mitigate negative impacts and strengthen workers’ mental health ^[27]^.

Supervisor support emerges as an important protective factor, as leadership grounded in support, fairness, and respect may prevent harmful practices such as bullying and workplace violence ^[17]^. Investments in healthy interpersonal relationships and psychosocial resources are essential. The interaction between supervisors and teams should be further evaluated and strengthened as a strategy to promote well-being at work ^[17]^.

Although this review identified relevant aspects related to mental health at work, it has limitations. The included studies focused predominantly on public hospitals and health professionals, particularly physicians and nursing staff, with limited representation of administrative and technical workers. In addition, the search included only one psychology database and did not cover databases specific to public administration. Further studies are recommended to expand the evidence base.

Nevertheless, the findings demonstrate the significant presence of workplace harassment and violence in the public sector and reinforce the need for interventions aimed at promoting worker health.

## CONCLUSIONS

This study demonstrated the presence of multiple forms of workplace harassment and violence in the public sector, with greater exposure among health professionals, particularly physicians and nursing staff. Verbal violence emerged as the most frequent form in public workplace settings, followed by physical violence and sexual harassment. The prevalence of harassment and violence was significantly higher in the public sector compared with the private sector.

Factors associated with increased susceptibility to occupational violence included night shift work, being married, female sex, and employment in emergency settings. Managerial support was identified as a favorable element with a potential protective effect in addressing these situations.

There is a need to develop and strengthen policies and guidelines aimed at promoting workplace safety, including institutional protocols for prevention and management of violence. Interventions focused on improving interpersonal relationships and the use of psychosocial resources also showed promise in some contexts. Mental health risks resulting from workplace harassment and violence should be incorporated into intervention and prevention strategies, including suicide prevention.

Overall, literature indicates that addressing workplace harassment and violence among public sector workers requires coordinated action involving managers, public policies, and educational programs, with emphasis on preventive measures and the promotion of healthier and more balanced work environments.

## Data Availability

The data supporting the findings of this study are available within the article.
